# Genomic differentiation within East Asian *Helicobacter pylori*

**DOI:** 10.1099/mgen.0.000676

**Published:** 2022-02-21

**Authors:** Yuanhai You, Kaisa Thorell, Lihua He, Koji Yahara, Yoshio Yamaoka, Jeong-Heon Cha, Kazunari Murakami, Yukako Katsura, TEAMHp, Ichizo Kobayashi, Daniel Falush, Jianzhong Zhang

**Affiliations:** ^1^​ State Key Laboratory of Infectious Disease Prevention and Control, Collaborative Innovation Center for Diagnosis and Treatment of Infectious Diseases, National Institute for Communicable Disease Control and Prevention, Chinese Center for Disease Control and Prevention, Beijing, PR China; ^2^​ Department of Infectious Diseases, Sahlgrenska Academy, University of Gothenburg, Sweden; ^3^​ Department of Clinical Microbiology, Sahlgrenska University Hospital, Västra Götaland 12 Region, Gothenburg, Sweden; ^4^​ Antimicrobial Resistance Research Center, National Institute of Infectious Diseases, Tokyo, Japan; ^5^​ Department of Environmental and Preventive Medicine, Oita University Faculty of Medicine, Oita, Japan; ^6^​ Department of Oral Biology, BK21 Plus Project, Yonsei University College of Dentistry, Seoul, Republic of Korea; ^7^​ Department of Gastroenterology, Faculty of Medicine, Oita University, Oita, Japan; ^8^​ Primate Research Institute, Kyoto University, Inuyama, Japan; ^9^​ TEAMHp (Team for East Asian Genomics of Helicobacter pylori)#; ^10^​ I2BC, University of Paris-Saclay, Gif-sur-Yvette, France; ^11^​ Department of Infectious Diseases, Kyorin University School of Medicine, Mitaka-shi, Tokyo, Japan; ^12^​ Department of Computational Biology and Medical Sciences (formerly Department of Medical Genome Sciences), Graduate School of Frontier Sciences, University of Tokyo, Tokyo, Japan; ^13^​ Institute of Medical Science, University of Tokyo, Minato-ku, Tokyo, Japan; ^14^​ Research Center for Micro-Nano Technology, Hosei University, Koganei-shi, Tokyo, Japan; ^15^​ The Center for Microbes, Development and Health, Key Laboratory of Molecular Virology and Immunology, Institut Pasteur of Shanghai, Chinese Academy of Sciences, Shanghai, PR China

**Keywords:** bacterial pathogenesis, flagellin glycosylation, gastric cancer, fixation index, multidrug efflux pump, outer membrane protein, population genomics

## Abstract

The East Asian region, including China, Japan and Korea, accounts for half of gastric cancer deaths. However, different areas have contrasting gastric cancer incidences and the population structure of *Helicobacter pylori* in this ethnically diverse region is yet unknown. We aimed to investigate genomic differences in *H. pylori* between these areas to identify sequence polymorphisms associated with increased cancer risk. We analysed 381 *H *. *pylori* genomes collected from different areas of the three countries using phylogenetic and population genetic tools to characterize population differentiation. The functional consequences of SNPs with a highest fixation index (Fst) between subpopulations were examined by mapping amino acid changes on 3D protein structure, solved or modelled. Overall, 329/381 genomes belonged to the previously identified hspEAsia population indicating that import of bacteria from other regions of the world has been uncommon. Seven subregional clusters were found within hspEAsia, related to subpopulations with various ethnicities, geographies and gastric cancer risks. Subpopulation-specific amino acid changes were found in multidrug exporters (*hefC*), transporters (*frpB-4*), outer membrane proteins (*hopI*) and several genes involved in host interaction, such as a catalase site, involved in H_2_O_2_ entrance, and a flagellin site mimicking host glycosylation. Several of the top hits, including *frpB-4*, *hefC*, *alpB*/*hopB* and *hofC,* have been found to be differentiated within the Americas in previous studies, indicating that a handful of genes may be key to local geographic adaptation. *H. pylori* within East Asia are not homogeneous but have become differentiated geographically at multiple loci that might have facilitated adaptation to local conditions and hosts. This has important implications for further evaluation of these changes in relation to the varying gastric cancer incidence between geographical areas in this region.

## Data Summary

All supporting data have been provided within the article or through supplementary data files. Public genome data were retrieved from the National Center for Biotechnology Information GenBank (Tables S2 and S3, available in the online version of this article). Newly sequenced genomes were deposited into GenBank with BioProject number PRJNA482300.upplementary material can

**Impact Statement**

East Asia, especially China, Japan and Korea, has the largest gastric cancer burden in the world. *Helicobacter pylori* in this region were thought to be homogeneous and uniformly highly virulent. However, although the incidence of gastric cancer shows considerable variation within the region, little is know about the population structure of *H. pylori *. Our whole-genome analysis implies that there is substantial regional variation within East Asian *H. pylori* subpopulation hspEAsia, including differentiation between low and high incidence regions. hspEAsia strains are differentiated geographically at a handful of genes that have facilitated adaptation to local conditions, including several genes implicated in similar studies of local differentiation within the Americas. Our analysis shows that genetic variation in *H. pylori* may be associated with gastric cancer incidence, both through direct and indirect causation.

## Introduction

*Helicobacter pylori* has co-evolved with human beings for at least 100 000 years and has strong association with the occurrence of gastric (stomach) cancer [1]. *H. pylori* are transmitted most effectively within households and have therefore experienced low migration rates compared to many other members of the human microbial flora. As a result, we can expect the pattern of differentiation to reflect historical human-migration patterns. The population structure of *H. pylori* worldwide has been classified into seven major groups that indeed correlate with ancient human migrations [2], of which the hpEastAsia includes at least three subgroups: hspEAsia, hspIndigenousAmerica [3] and hspMaori. The hspEAsia subgroup is thought to be ubiquitous within East Asian countries with high gastric cancer incidence, including China, Japan and Korea. These countries together make up 1/5 of the world population but account for half the global mortality from the disease [4, 5]. The hspEAsia strains have documented higher virulence than other subpopulations and have diverged from the Western strains in several proteins including virulence factors [6, 7]. Many genes have also diverged *within* this region [6].

**Fig. 1.**
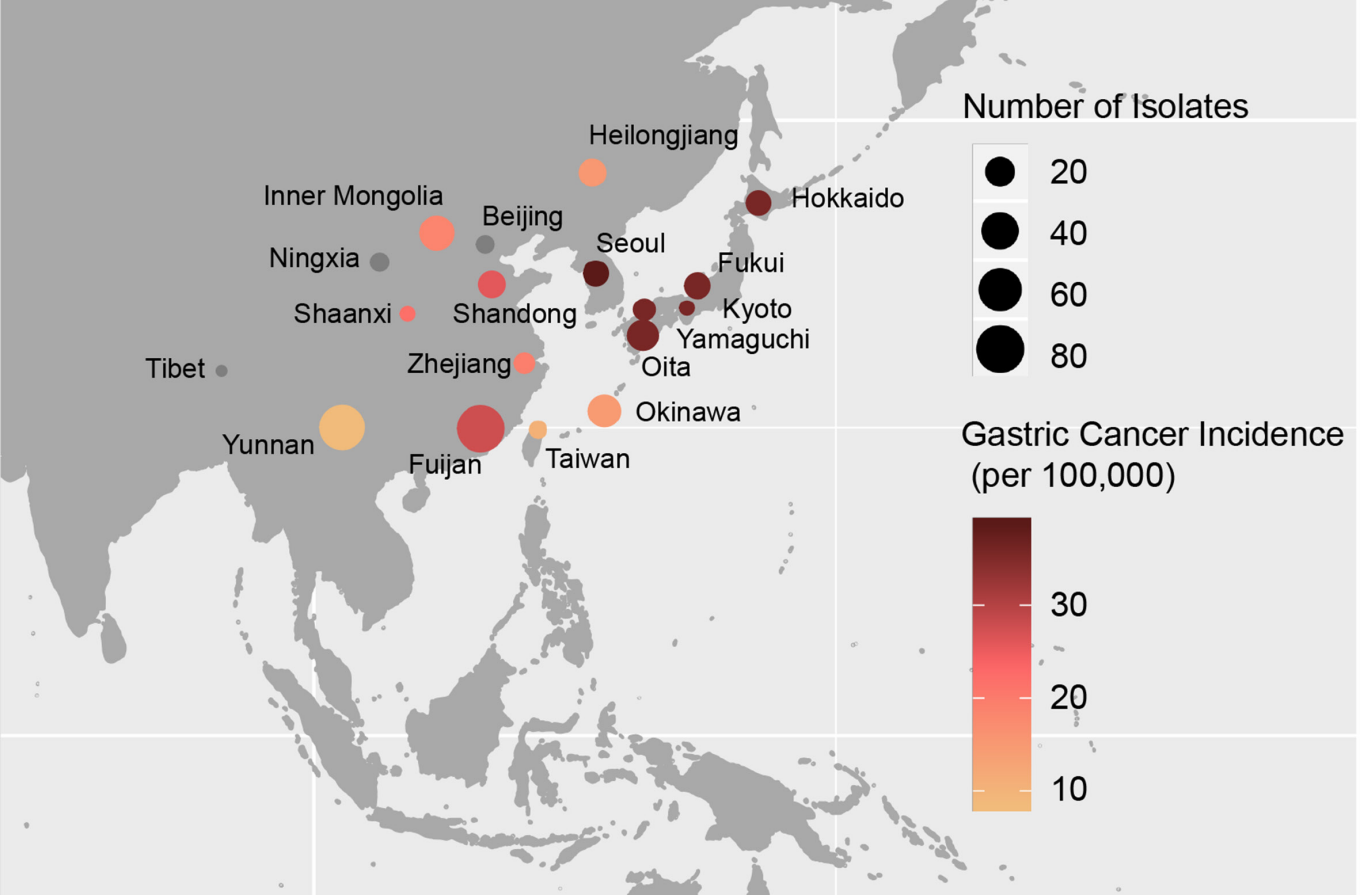
Geographic distribution of the East Asian *H. pylori* strain dataset of this study. Shade of the circle fill indicates gastric cancer incidence. Grey fill indicates missing incidence data. All are age-standardized gastric cancer rates from 2014 by world population except that for Okinawa, only the data from 2016 is available.

The prevalence of gastric cancer shows geographic and ethnic variations also within East Asia. Some north and southeast areas of China such as Fujian have higher incidence [8], whereas some west regions, such as Yunnan, have low incidence (Fig. 1) [9, 10]. China also shows diverse distribution of population ethnicities; in southwest Yunnan province, more than 20 ethnicities exist. South Korea has been reported with the highest incidence in the world [11, 12] and similar is true for the Japanese main islands (including Hokkaido) [13], while in Okinawa, where the ethnic composition is different, the incidence is low [13, 14]. Previous studies [8, 15] on the high-risk regions have suggested that diet, lifestyle and *H. pylori* properties may contribute to the high risk. However, despite the complex human migrations and evolutionary history in these areas, variation of *H. pylori* within the hspEAsia subpopulation has been poorly explored.

In the present work, we analysed a collection of genomes of *H. pylori* strains from various places in these three countries. Our phylogenetic and population genetic analysis revealed presence of pronounced regional and ethnical population structure within hspEAsia, and specific sequence differences differentiating these subpopulations. Most of these highly region-specific variants were found on proteins involved in host interaction. Furthermore, placement of the variants on protein structure provided insight into molecular mechanisms underlying regional adaptation.

## Methods

### Strain collection across East Asia

We collected 357 *H *. *pylori* isolates from China, Japan and South Korea, isolated between 1999 and 2018, including 11 provinces of China and six regions of Japan (Tables S1,S2, Fig. 1). We used data from 2014 to show regional risk levels in China and compared it with data from Japan and Korea [8, 9, 11–14]. Our sample of 77 Yunnan isolates includes strains from four ethnic minorities and the Han majority individuals.

### Genome sequencing

Genomic DNA was extracted using the Qiagen DNeasy Mini Kit and genomes were sequenced using Illumina or PacBio sequencers. For Illumina sequencing, paired-end libraries were created and sequenced using the Illumina Hiseq and Miseq platform. For PacBio sequencing, quality assessment of genomic DNA, SMRTbell library preparation and data evaluation were performed, and sequenced using the PacBio RS II platform. Sequences were deposited into GenBank with BioProject number PRJNA482300. Combining publicly available genomes (available in January 2018 when the study started) with these newly sequenced genomes, we collected a dataset consisting of 381 *H *. *pylori* genomes for further analysis (Table S2).

### Genomic comparison

We used snippy [16] to perform a whole-genome alignment with XZ274 as the reference genome and extracted 225 942 variable core-genome sites. For the phylogenetic analysis, the dataset of 381 strains were combined with 1–3 reference sequences for each of the major *H. pylori* populations (Table S3).

### Population structure and phylogenetic analysis

The concatenated whole-genome SNPs and coordinates were used to prepare the haplotype file for ChromoPainter and fineSTRUCTURE analyses following the instructions from website (http://www.paintmychromosomes.com). In total, 13 highly clonal sequences were removed prior to the analysis, resulting in a comparison of 370 genomes. Using each genome as both donor and recipient haplotypes, we used ChromoPainter to calculate the number of genetic chunks exported from a donor to a recipient and generated a co-ancestry matrix. Then the co-ancestry matrix file was imported into fineSTRUCTURE by setting the burn-in and Markov chain Monte Carlo (MCMC) chain of 100 000 iterations to generate clusters for all the individual strains [17, 18]. We also generated a phylogenetic tree using FastTree (http://www.microbesonline.org/fasttree) [19], which was labelled using iTol (https://itol.embl.de) [20].

### Definition of subgroups and calculation of Fst between subgroups

Subgroups were defined according to the population structure analysed by fineSTRUCTURE (the left-side tree). We named the subgroups assigned by fineSTRUCTURE as ‘Sg’ and also according to the geographic origin of the majority of a clade (Table S2). This criterion for subgroup definition is relatively a rough classification. To identify the SNPs attributed to the divergence of subgroups more accurately, we used a more stringent criterion for a further definition of the subgroups. Only those isolates assigned into a singular cluster in the fineSTRUCTURE tree were defined to form a subset of a subgroup and used for Fst calculation. For example, for the large sampling areas such as China southwest isolates, only those from Yunnan Mosuo and Pumi ethnicities, which clustered into a singular clade with low levels of admixture/recombination, were defined as a subgroup, ‘YunnanMP’. For China southeast, only those from Fujian Changle that clustered into a singular clade were defined as a subgroup, ‘Fuijan’. Subgroups with mixed ethnicities or high levels of admixture were not used in the calculations.

For each SNP site, we calculated a fixation index (Fst) using PopGenome R package [21] for each subgroup such that Fst (sg1)=pairwise calculation of Fst of sg1 versus all other subgroups. We also compared isolates from China with those from Japan and South Korea and the two lower-incidence regions, Yunnan and Okinawa, versus high incidence regions (the remainder of hspEAsia strains).

### Mapping high-Fst SNPs on protein structure

We located each of the SNPs with the highest Fst values on the reference genome of XZ274, a Tibetan strain, to identify its gene and effect on amino acid sequence. If the gene was missing or atypical in this strain, we used strain F57, a Japanese strain, or 26 695, a European strain, instead as shown in Table S4. We mapped the amino acids on solved *H. pylori* protein structure or on protein structure homology-modelled by SwissModel and its repository for 26695 (https://swissmodel.expasy.org/repository). We analysed and presented them by PyMOL [22].

## Results

### East Asian *H. pylori* population structure is associated with geography and host ethnicity

**Fig. 2.**
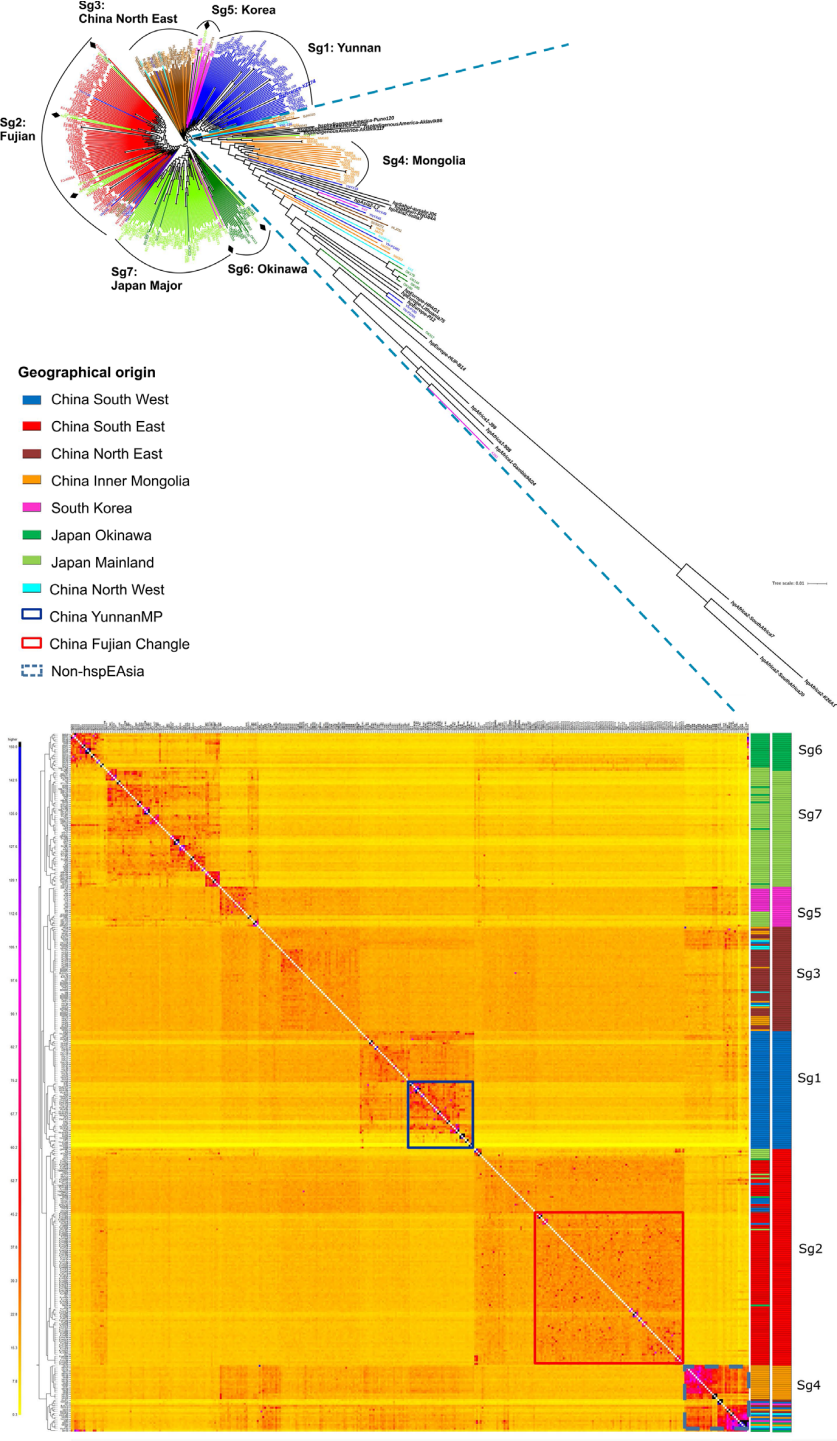
Phylogenetic and population genetic analysis of East Asia *H. pylori* strains. (a) Phylogenetic tree of East Asia genomes combined with reference sequences from global *H. pylori* populations. Diamonds indicate Japanese and Korean isolates that are dispersed in other subgroups. (b) Co-ancestry matrix of East Asia isolates calculated using fineSTRUCTURE after removing isolates with highly clonal relatedness. The tree inferred on the left of the co-ancestry matrix indicates the relationships between groups with distinct ancestry profile as inferred by fineSTRUCTURE. On the right side, the first column shows the geographic origin of each isolate. The second column indicates each subgroup with a distinct colour.

To analyse the genetic structure of *H. pylori* in China, Japan and South Korea, we constructed a phylogenetic tree (Fig. 2a) and clustered the strains using fineSTRUCTURE [17, 18] (Fig. 2b). The two methods gave broadly concordant clustering and indicated differentiation at multiple scales. By including reference genomes from the other main *H. pylori* populations, we found that the 52 most differentiated strains do not belong to hspEAsia and have had distinct evolutionary histories. One main cluster of these comprised isolates from individuals of Mongolian ethnicity (Sg4, Fig. 2b), which in the tree grouped between the references of hspIndigenousAmerica (previously called hspAmerind) and hpAsia2 populations. We also identified a cluster of Okinawan strains diverging after hpEurope, and before hpAsia2 and hpEastAsia (Fig. 2a), also shown in the lowest line of the co-ancestry matrix (Fig. 2b). This likely corresponds to group C in MLST and STRUCTURE analysis of Okinawan strains [23]. Because these populations are best analysed in the context of broader regional variation, we focused our remaining analysis on variation within the 329 hspEAsia isolates in our sample.

HspEAsia isolates are relatively homogeneous but show fine-scale differentiation that is strongly correlated with geography. The majority of Japanese isolates clustered together (Sg7), with Okinawan hspEAsia strains forming a distinct subpopulation (Sg6). The latter likely corresponds to group A in MLST [23]. Two strains from Hokkaido cluster with the Sg6 Okinawan strains. This is likely related to the evolutionary structure of Japanese people and of the Okinawa people [24]. The native ethnicity remains in Hokkaido (as Ainu ethnicity) and in Okinawa.

South Korean isolates also form a distinct subpopulation, which cluster together with isolates from the Northeast of China, concordant with its geographic location. A few Japanese strains are in the Korean cluster while a few Korean strains are in the main Japanese cluster and branching around the same time as the Okinawa cluster (Fig. 2a,b). This may reflect immigration from Korea to Northern Kyushu and Okinawa. Within China, there is clear differentiation between the Northeast, Southeast and Southwest areas. Within the Southwest, a subpopulation containing strains from Yunnan Mosuo and Pumi ethnicities could also be observed. A Tibetan isolate clustered with the Pumi population, consistent with their migration and mixture history. Within the Southeast, there was also a subpopulation (Sg2), specific to Fujian Changle, a region of high gastric cancer incidence (Fig. 2, table S1). The Southeast cluster also includes multiple clusters of Japanese/Okinawa strains (Fig. 2a,b) likely reflecting later immigration to Japan/Okinawa.

#### Many genetic variants show strong differentiation by subpopulations

In order to explore the genetic basis of local differentiation, we calculated fixation index, Fst, between the hspEAsia subpopulations identified by fineSTRUCTURE. To obtain more reliable SNPs associated with subgroup separation, we used the YunnanMP subcluster of Sg1 and Fujian subcluster of Sg2, along with the remaining five subgroups defined by the fineSTRUCTURE analysis. For most of the subpopulations, more than 99 % of SNPs were weakly differentiated, with Fst less than 0.3 (Fig. S1). The Korean subpopulation had a smaller sample size and showed the weakest Fst values. We defined clustered nucleotide polymorphisms (CNPs) as occurring when two or more SNPs with high Fst were found in the same gene. CNPs likely result from the co-inheritance of different SNPs on the same gene fragment.

To functionally interpret differentiation between the populations, we focused on the SNPs with the top 20 Fst values in each subgroup and then removed SNPs with Fst <0.5. This resulted in a list of 56 genes, several of which occurred more than once (Table S4, bold, summarized in Table 1). In the main text, we focus on the genes with the strongest evidence for differentiation. Eight of these had one SNP with Fst >0.6 and appeared in at least two top 20 lists in different pairwise comparisions. Another four had at least two SNPs with Fst >0.6. Of these, HofC has multiple non-synonymous SNPs with Fst up to 0.86, marking it out as also being a particularly strong candidate for being differentiated by natural selection (Table 2).

**Table 1.** High Fst SNPs distributed in each hspEAsia subgroup and involved genes^∗^

Subgroup	Gene	Annotation	Fst	SNP coordinate
Sg1 (YunnanMP)	*hefC*	Inner pump of a multidrug efflux system	0.891	827 628
	*porB*	Pyruvate-ferredoxin oxidoreductase subunit beta	0.800	1 175 532
	*hefC*	Inner pump of a multidrug efflux system	0.757	827 633
	*flaA*	Flagellin A	0.748	833 437
	*gltA*	Citrate synthase	0.731	1 418 093
	*tlpD*	Chemotaxis sensor	0.716	836 384
	*katA*	Catalase	0.714	507 178
	*gltA*	Citrate synthase	0.706	1 418 291
	*gltA*	Citrate synthase	0.706	1 418 293
	*flaA*	Flagellin A	0.705	833 501
	*frpB-4*	TonB-dependent outer membrane Ni importer	0.694	1 536 189
	*oppD*	Oligopeptide permease ATPase protein	0.669	283 539
	*oppD*	Oligopeptide permease ATPase protein	0.663	283 540
	*porB*	Pyruvate-ferredoxin oxidoreductase subunit beta	0.649	1 175 543
	*hopI*	Outer membrane protein HopI	0.638	1 220 823
	*gltA*	Citrate synthase	0.626	1 418 759
Sg2 (Fujian)	*hofC*	Outer membrane protein involved in adhesion and diffusion of cations including antibiotics	0.855	496 438
	*hofC*	Outer membrane protein involved in adhesion and diffusion of cations including antibiotics	0.850	496 437
	*hofC*	Outer membrane protein involved in adhesion and diffusion of cations including antibiotics	0.800	496 436
	*katA*	Catalase	0.756	507 441
	*hopB/alpB*	Outer membrane protein, Omp21	0.704	983 839
	*flaA*	Flagellin A	0.651	833 026
Sg3 (China North East)	*frpB-4*	TonB-dependent outer membrane Ni importer	0.752	1 536 144
	*frpB-4*	TonB-dependent outer membrane Ni importer	0.752	1 536 145
	*frpB-4*	TonB-dependent outer membrane Ni importer	0.738	1 536 086
	*frpB-4*	TonB-dependent outer membrane Ni importer	0.738	1 536 093
	*frpB-4*	TonB-dependent outer membrane Ni importer	0.713	1 536 091
	*frpB-4*	TonB-dependent outer membrane Ni importer	0.706	1 535 959
	*frpB-4*	TonB-dependent outer membrane Ni importer	0.701	1 535 957
	*frpB-4*	TonB-dependent outer membrane Ni importer	0.701	1 535 965
	*frpB-4*	TonB-dependent outer membrane Ni importer	0.637	1 535 958
Sg5 (Korea)	*hypC*	hydrogenase expression/formation protein	0.652	959 689
Sg6 (Okinawa)	*rhoD*	Rhodanese, a cyanide-detoxifying enzyme	0.646	1 299 845
Sg7 (Japan_Major)	*frpB-4*	TonB-dependent outer membrane Ni importer	0.658	1 536 096

^∗^ Criteria for showing high Fst of each subpopulation in this table is based on the following:

Fst > 0.6.

Sg4 (Mongolia) is exclued from the table as it does not belong to the hspEAsia subgroup.

SNPs of high/low and China_All are not listed because they were specific comparisons, not representing subpopulations.

**Table 2.** Genes with a SNP with Fst >0.5 in the top 20 lists

Criterion	Gene	Locus tag	Figure	Category	Annotation	Residue function
Fst >0.6+ top 20 in different pairwise comparisons	*hefC*	HP0607	3(b)	Efflux pump	Inner membrane component of the multidrug HefABC efflux pump	Channel entrance
	*frpB-4*	HP1512	4(a)	TonB-dependent importer	Outer membrane nickel importer	Ligand binding
	*flaA*	HP0601	6(b)	Motility	Flagellin A of flagella, involved in immune system evasion	Glycosylation for host mimicry and more
	*katA*	HP0875	6(a)	Sensor	Catalase sensing and destroying H_2_O_2_	Channel entrance; Dimer-dimer interface
	*hopI*	HP1156		Outer membrane protein	Outer membrane protein HopI	
	*hopB/alpB*	HP0913		Outer membrane protein	Hop family adhesin HopB/AlpB/Omp21.	
	*tlpD*	HP0599	6(c)	Sensor	Chemotaxis sensor sensing and destroying HOCl	Active site
	*exbB-2*	HP1339	S3C	TonB-dependent importer	Energizer/motor in the inner membrane driven by proton	Channel entrance
Fst >0.6 two or more SNPs	*hofC*	HP0486		Outer membrane protein	Hof family protein implicated in adhesion and antibiotics diffusion	
	*porB*	HP1111		Energy metabolism	Subunit of pyruvate:ferredoxin oxidoreductase, part of the microaerophilic metabolic pathway leading to acetyl~CoA	Subunit interaction
	*gltA*	HP0026	5(b)	Energy metabolism	Citrate synthase, the first enzyme in TCA cycle incorporating acetyl~CoA	Subunit interaction
	o*ppD*	HP0250	5(a)	Importer	Oligopeptide ABC transporter, ATPase subunit	Cofactor binding
Fst >0.5	o*mpA-18*	HP1125		Outer membrane protein	OmpA family peptidoglycan-associated lipoprotein	Ligand binding
	*fixP*	HP0147	S4C	Energy metabolism	Subunit of cytochrome c oxidase in aerobic respiration	Channel entrance
	*hypC*	HP0899			Hydrogenase expression/formation protein	(Synonymous change)
	*hefD*	HP0971	3(c)	Efflux pump	Outer membrane component of the HefABC multidrug efflux pump	Protein binding
	*copA*	HP1503	S2E	Exporter	Copper(I) exporter	Protein stability
	*mscS-1*	HP0284		Sensor	Mechano-sensor sensing tension in the membrane	Scaffolding in the periplasm
	*metQ*	HP1564	S2D	Importer	d-methionine ABC transporter, methionine-binding subunit	Ligand binding
	*frpB-1*	HP0876	S3A	TonB-dependent importer	Outer membrane haem importer	Ligand binding
	*fecA-1*	HP0686	S3B	TonB-dependent importer	Outer membrane iron(III) dicitrate importer	Ligand binding; Channel (plug)
	*exbD-2*	HP1340	S3C	TonB-dependent importer	Inner membrane energizer/motor driven by proton	Proton channel entrance
	*hpaA*	HP0797	S3F	Outer membrane protein	Neuraminyllactose-binding hemagglutinin	Subunit interaction
	o*mp*	HP0358		Outer membrane protein	Putative outermembrane protein	
	*panD*	HP0034	S4A (i)	Micronutrient synthesis	Vitamin B5 synthesis	Interaction between cleaved peptides
	*bioD*	HP0029		Micronutrient synthesis	Vitamin B7 synthesis	
	*tgt*	HP0281	S4A (iii)	Micronutrient synthesis	Q-base synthesis; Q-base on tRNA affects translation accuracy	Active site
	*rhoD*	HP1223	S4B	Detox	Rhodanese detoxifying cyanide generated in microbiome	
	*rkiP*	HP0218	S2B	Oncoprotein	Mimic of human RKIP tumour suppressor	Interaction with signal peptide
	*hcpX*	–	S2A	Effector	SLR family with repeated alpha-helix pairs	Human protein binding
	*dsbI*	HP0595	S3D	Secretion	S-S formation	Active site
	*jag*	HP1451		Secretion	Regulator of VirB11/Cag-alpha gate of Cag secretion system	Cofactor binding
	*lpxE*	HP0021	S2C	Membrane lipid modifier	Lipid A 1-phosphatase to hide it from the innate immune response	Substrate binding; active site
	*cfaS*	HP0416	S3E	Membrane lipid modifier	Cyclopropane-fatty-acyl-phospholipid synthase for acid protection	Substrate binding
	*fur*	HP1027	S4D	Transcription factor	Regulator of Fe/Ni import, redox balance and acid response	Cofactor binding

To predict the effect of differentiated variants on *H. pylori* biology and pathogenesis, we functionally annotated the genes and interpreted the impact of differentiated amino acids on the protein structures, solved or based on homology modelling. The majority of genes containing the most differentiated SNPs could be grouped into four major categories; (i) transporters, (ii) outer membrane proteins, (iii) metabolism and (iv) host interaction.

### Transporters

#### The Hef multidrug efflux pump

**Fig. 3.**
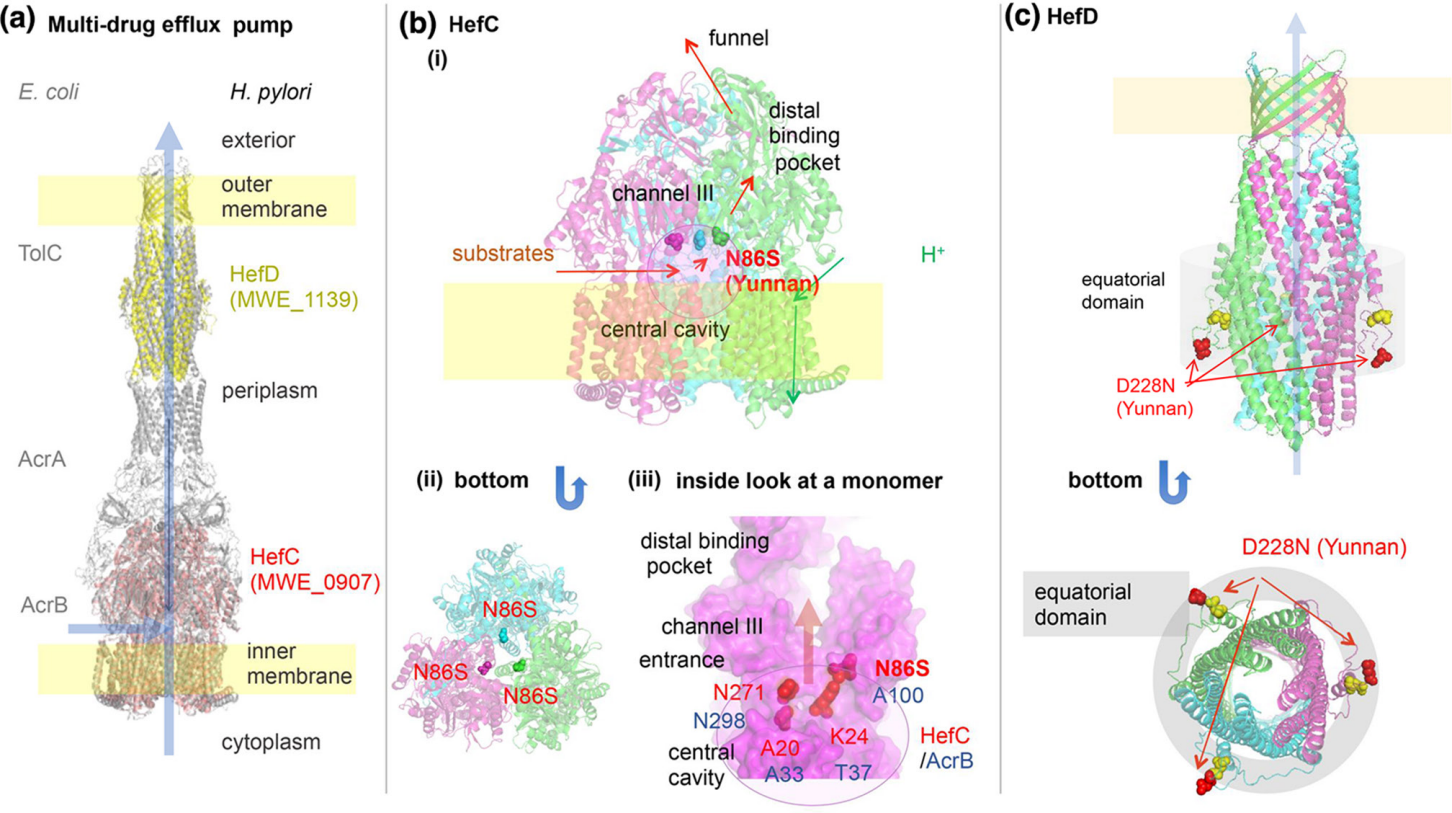
Inferred structure and differentiated amino acid sites of multidrug efflux pump related proteins. (a) TolC-AcrA-AcrB of *E. coli* and its *H. pylori* homologues. (b) HefC (MWE_0907), modelled on PDB 3W9I (MexB of *Pseudomonas aeruginosa *). (iii) HefC modelled on PDB 3AOD (AcrB of *E. coli*). Yunnan-specific N86S is at the entrance of channel III. (c) HefD (MWE_1139), modelled on PDB 5BUN (ST50 from *Salmonella enterica* subsp. enterica serovar Typhi). Yellow spheres indicate Mongol-differentiated E219D.

*H. pylori* carries four gene clusters that each encode a set of RND superfamily of multidrug efflux pump corresponding to TolC-AcrA-AcrB of *E. coli* (Fig. 3). They pump out endogenous bile salts and ceragenins as well as various antibiotics [25–27]. The single most differentiated SNP in our analysis is in *hefC* (HP0607) in the YunnanMP subpopulation, with an Fst=0.89. This N86S is also the most differentiated between the lower cancer incidence regions (Yunnan and Okinawa) and the remainder. This residue corresponds to the gate to channel III for planar aromatic cations in the *E. coli* homologue [28] and the regional adaptation may therefore remodel this gate to export chemicals of this type at different ratios. Also, the outer component of the efflux pump, HefD, showed YunnanMP specific residues in the equatorial domain that may be involved in the interaction with the inner component to open the aperture.

#### The TonB-dependent nickel importer FrpB-4

**Fig. 4.**
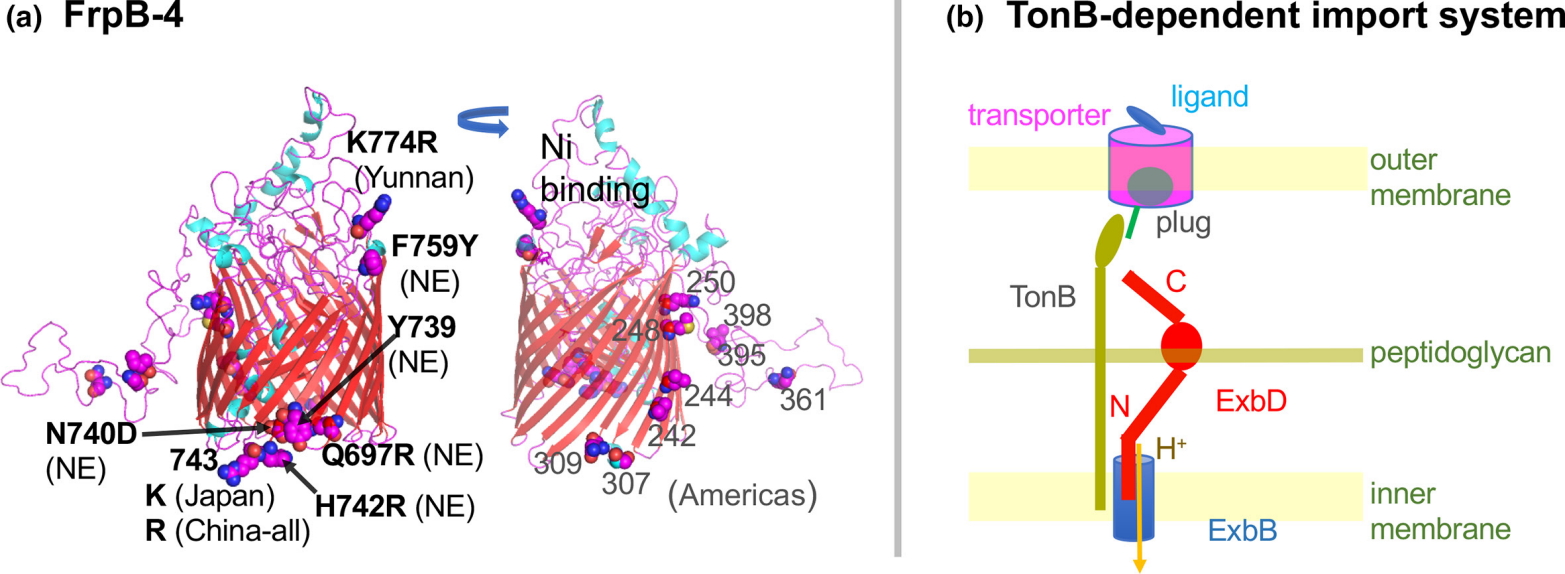
(a) FrpB-4 (MWE_1700) modelled on PDB 4AIQ (FrpB of *N. meningitidis*). The left figure shows East Asia-specific sites. NE means north east China. The right one shows Americas-specific sites. (b) TonB-dependent transport system. When the transporter in the outer membrane catches a ligand, it pushes out its plug for TonB to pull and open the channel. This movement of TonB is energized by ExbBD motor in the inner membrane driven by H^+^ flow.

A gene containing multiple highly differentiated SNPs, especially in North Eastern China, is *frpB-4* (HP1512), encoding an outer membrane transporter of nickel of some form [29]. It is a member of TonB-dependent transporter family, which forms a trimer of 22-stranded beta barrels each filled with a ‘plug’ (Fig. 4a,b). *H. pylori* reference strain 26695 carries four *frpB* homologues: *frpB-1* (HP0876), *frpB-2/3* (HP0916/5) and *frpB-4* (HP1512).

Ligand binding lets TonB change the conformation of the plug, which opens a channel, a process energized by the ExbBD proton-driven motor [30] (Fig. 4b). Four China North East-specific Fst sites cluster (Fig. 4a), with three sites (739, 740, 742) presumably representing a CNP. The next residue (743) distinguishes between Japan (K) and China (R). Northeast China-differentiated F759Y (F for the remainder and Y for Northeast China) and YunnanMP-differentiated K774R are situated above the barrel in the model and may interact with the ligand. Various regions of the Americas show region-specific amino acid changes in other areas of the protein [31], out of which three are predicted to be in the decoy loop. Taken together, these changes may affect nickel transport and, consequently, urease activity, since the urease enzyme requires nickel for acid acclimation [32]. The changes could be related to regional differences in host nickel metabolism and stomach acidity.

### Other transporters

Hof proteins (* Helicobacter *-specific outer membrane protein family [33]) are 18-stranded β-barrels homologous to Occ family of *Pseudomonas* and *Campylobacter jejuni* MOMP (major outer membrane protein) involved in passive diffusion of cations including antibiotics and in adhesion [34–36]. HofC (HP0486), required for *H. pylori* colonization in mice [37], contains the most differentiated SNPs in Fujian, a region of high cancer incidence with Fst=0.86. The gene is highly variable in global strains and shows many America-differentiated SNPs and region-differentiated SNPs within the Americas [31], within one narrow region. Fujian-differentiated D186S in HP0486 (166 in MWE_0556) lies at a distance from them. Fujian-differentiated V9A is in its signal peptide.

**Fig. 5.**
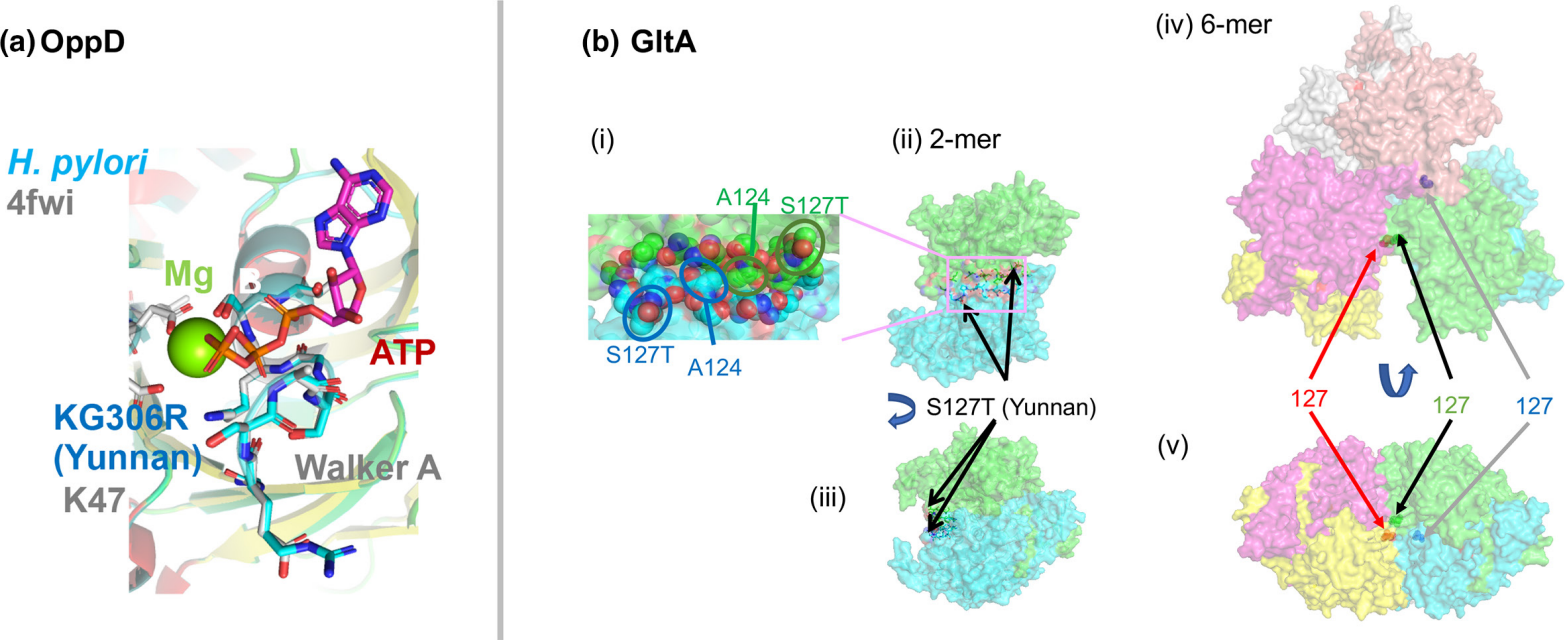
(a) OppD (MWE_0327), ATP-binding subunit of an oligopeptide ABC transporter as modelled on PDB 4FWI (* Thermoanaerobacter tengcongensis *). (b) Citrate synthase, GltA (i) (ii) (iii) MWE_1570 modelled on PDB 3MSU (* Francisella tularensis* homolog). (iv) (v) On PDB 2h12 (* Acetobacter aceti *).

OppD (HP0250), the cytoplasmic subunit of the oligopeptide ABC transporter, has YunnanMP-differentiated KG306R within the ATP binding Walker motif A (Fig. 5a).

### Outer-membrane proteins

HopB/ AlpB/ Omp21 (HP0913), an adhesin of the Hop family required for colonization, carries Fujian-differentiated N289D and N286H. According to a previous study, 24 polymorphic sites within 49 bp in AlpB are enriched for Asian ancestry in hspEuropeColombia and 32 polymorphic sites within 65 bp were enriched for Asian ancestry in hspAfrica1Nicaragua populations [31].

Another member of the Hop family of outer-membrane proteins, HopI (HP1156), has a site (467) that distinguishes between Japan (H) and China-all (D) and YunnanMP-differentiated V633L.

### Central metabolism

The region-differentiated amino acid changes involve a handful of key metabolic enzymes.

Citrate synthase GltA (HP0026, MWE_1570), is the first enzyme in the TCA cycle catalysing the conversion of acetyl-CoA and oxaloacetate to citrate. Yunnan-differentiated S127T is located between the two identical monomers and is likely involved in their association as well as in dimer–dimer association to form a 6-mer (Fig. 5b). The differentiated SNP might change the quaternary structure. Mutation of A124 in this interface was found in experimental evolution in *E. coli* [38]. In addition to GltA, two other key metabolic enzymes, PorB (HP1111), a subunit of pyruvate:ferredoxin oxidoreductase, and FixP (HP0147, MWE_0216), a subunit of cytochrome c oxidase, had high Fst values in YunnanMP. PorB, a key enzyme in the microaerophilic metabolism of *H. pylori,* converts pyruvate to acetyl-CoA, the substrate of GltA, and FixP is a component in aerobic respiration. The Yunnan-differentiated residue is at the proton entrance (Fig. S4C). Together, these changes might affect the metabolic capacity of this regional *H. pylori* subpopulation.

### Host interaction

In addition to the genes listed above, region-specific non-synonymous variants are present in several genes that are annotated as known virulence or host interaction factors.

**Fig. 6.**
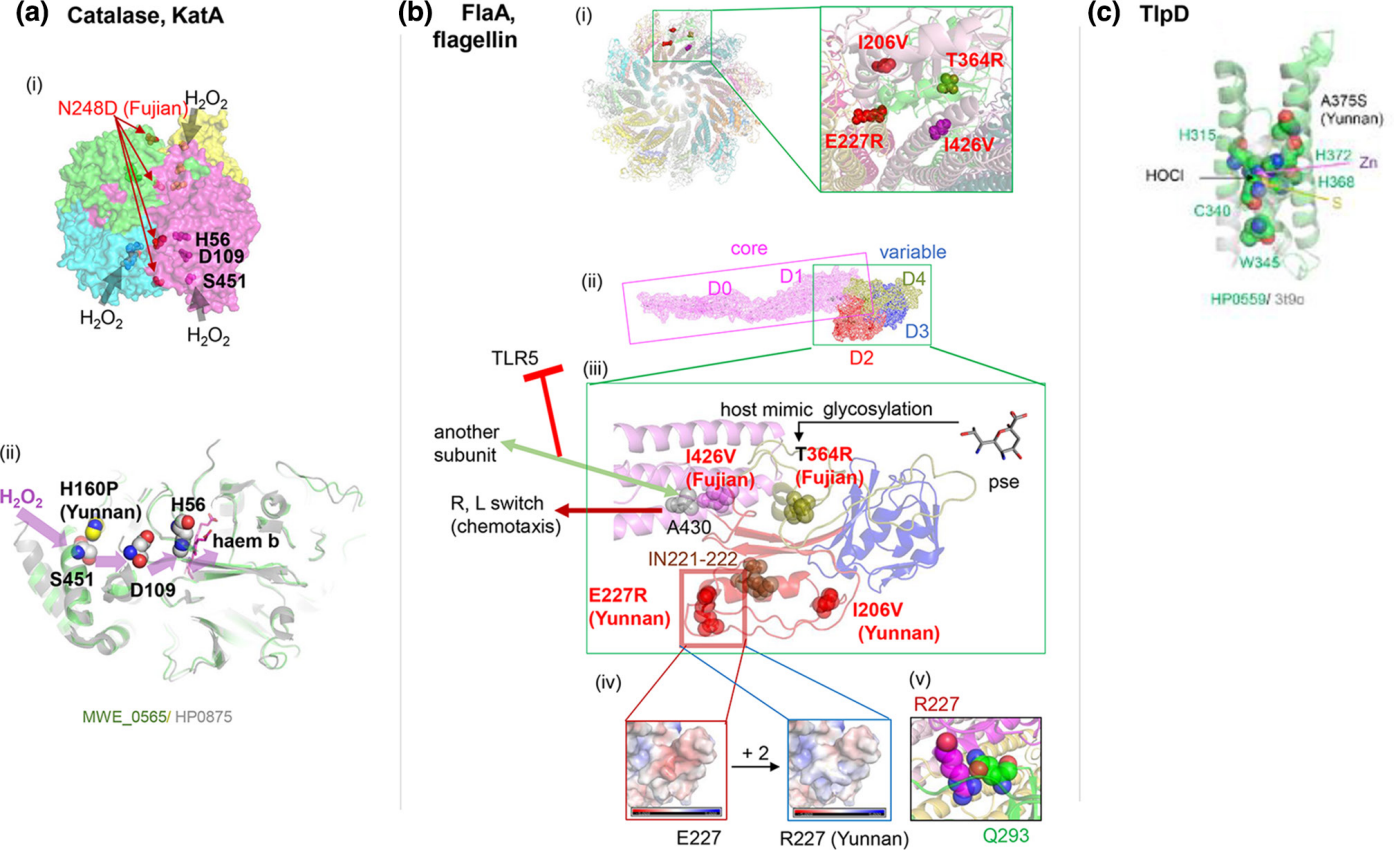
(a) Catalase KatA (HP0875, PDB 2A9E). (i) Dimer of dimer. (ii) Channel for H_2_O_2_.Yunnan-specific P160H by its entrance S451 drastically changes local conformation and surface electric charge (mutagenesis in PyMOL). (b) Flagellin, FlaA. (i) A view from the distal end of the 22-mer model of FlaA (MWE_0913) on G508A mutant of *C. jejuni* homologue (PDB 6×80) with four population-specific amino acid changes in two interacting monomers. (ii) Monomer with five domains. (iii) Population-specific amino acid changes. pse: pseudaminic acid. (iv) Surface electric charge change by E227R. (v) R227 interaction with a neighbouring monomer. (c) TlpD, chemotaxis receptor for HOCl. HP0559 modelled on PDB 3T9O, the regulatory CZB domain of DgcZ (*E. coli*).

Catalase KatA (HP0875) (Fig. 6a) detoxifies H_2_O_2_ generated by host immune cells. It also binds host vitronectin, thereby protecting against complement-mediated killing [39]. The preferred route for H_2_O_2_ is the channel S451-D109-H56-haem (Fig. 6a (ii)) [40]. YunnanMP-differentiated P160H by the entrance S451 drastically changes local conformation and surface electric charge. Fujian-differentiated N248D with −1 change in the electric charge takes place near the dimer–dimer interface (Fig. 6a (i)) likely changing their interaction.

The flagellar filament made of flagellin FlaA (HP0601) (Fig. 6b), is involved in motility, cell adherence and immune modulation. We have modelled it using the similar *C. jejuni* homologue [41]. The flagellin has rod-shaped domains forming a hydrophobic core, and the other domains decorating the surface of the filament are hypervariable. The flagellin is glycosylated by pseudaminic acid at several residues to stabilize the flagellum and to mimic host-cell surface, a way for the bacterium to modulate the immune response [42], but Fujian-differentiated T364R eliminates one of these sites (Fig. 6b (iii)). E227R drastically changes the surface electric charge and likely affects its interaction with a neighbouring monomer (Fig. 6b (iv)(v)). Another Fujian-differentiated residue 426 in the conservative core is next to residue 427, which is involved in evasion from TLR5-mediated innate immunity through subunit interaction [41]. Furthermore, residue 430 adjacent to 426 in the 3D structure is somehow involved in switching between R and L conformations for swimming/tumbling in chemotaxis in *Campylobacter* [43].

TlpD (HP0599, MWE_0916) (Fig. 6c) is a cytosolic chemotaxis sensor required for colonization. TlpD senses HOCl, an antimicrobial produced by neutrophils during inflammation [44]. HOCl oxidizes a conserved cysteine (C340) within a 3His/1Cys Zn-binding motif to inactivate chemo-transduction signalling. YunnanMP-differentiated A375S is right by this motif. Additional proteins in Table 2 are described in the Supplementary Material.

## Discussion

Analyses of *H. pylori* in East Asia have tended to emphasize their homogeneity and uniformly high virulence potential. A single subpopulation of the bacteria, hspEAsia, is prevalent in the region. The hspEAsia strains have been found to be invariably CagPAI positive, with the *cagA* gene containing the ABD EPIYA motif, which is thought to promote strong binding of the protein to SHP-2 [45]. Our large collection of genomes of *H. pylori* from multiple regions in China, Japan and Korea confirm these observations. Only a small number of these isolates, of which a majority are from Mongolia and Okinawa, belonged to other *H. pylori* populations. For the scope of this study, these were excluded from subsequent analyses. All but 13 of the total 381 isolates were *cagA* positive and 355 have the characteristic ABD EPIYA type, including 316 out of 329 hspEAsia isolates.

Our results add a layer of complexity to the picture of uniformity by demonstrating that there is differentiation of *H. pylori* strains of the hspEAsia subpopulation between regions in East Asia. Despite a large burden of gastric disease in the region, most *H. pylori* infections by hspEAsia are asymptomatic, and the gastric cancer incidence varies widely across the region, especially in China. A large part of these differences might be attributable to diet and environment. However, our results imply that there are also bacterial factors differentiating these regions, which may be significant for disease development, especially because the bacteria themselves can adapt to environmental conditions [6].

Geographic differentiation between populations accumulates progressively when migration rates between them are low. Further, adaptation of bacteria to differences in environmental conditions can greatly accelerate the process of differentiation in specific regions of the genome. Our results, in combination with a previous study of genetic variation within the Americas [31] suggest that there are a handful of loci that have undergone rapid differentiation in several regions*,* and therfore may be considered keys for host adaptation. These include the genes *frpB-4*, *hefC*, *alpB*/*hopB* and *hofC*.

Our strategy to identify geographically differentiated SNPs by dividing one population (hspEAsia) into minimal subpopulations, therefore strains with consistent population and strain labels, and comparing fixation index (Fst) site-by-site between these populations reveal numerous loci of differentiation (Table S4). Of these we discuss the 12 with the strongest evidence for being involved in local adaptation in more detail in the main text in this paper. Some of the region-specific SNPs are in genes encoding for proteins that have been implicated in host interaction and virulence in the narrow sense: attack by immune system (catalase, TlpD), host adhesion (HopB/AlpB and several outer membrane proteins), and host surface mimicry (flagellin FlaA). Several of the other genes are transporters that may have implications for antimicrobial resistance, or are involved in nutrient acquisition. These results suggest that various host-adaptive changes in many host-interaction proteins lead to population differentiation. A similar gene set was found when rapid genome changes were investigated in shorter-term, intra-body micro-evolution [46].

Epidemiological and experimental evidence suggests that iron-deficiency increases *H. pylori* virulence and risk of gastric cancer [47]. In our analyses we could see both in iron and nickel metabolism highlighted by regional changes. Apart from the above-mentioned *frpB-4,* genes encoding for the TonB motor proteins ExbB-2 and ExbD-2, haem transporter FrpB-1 [48] appear in the list of the 34 genes with Fst >0.5 and the central transcription factor ferric uptake regulator, Fur, had regional variants (Supplementary Material). The causal mechanism underlying this association is not clear but it plausibly reflects bacterial response to nutrient limitation. Simply put, it is possible that bacteria adopt more aggressive strategies in interacting with the host and its microbiome when iron and other metals such as nickel, which is necessary for urease function, is in short supply.

In other organisms, linkage means that high Fst regions often occur in large blocks, making it difficult to infer which sites are involved in local adaptation. However, *H. pylori* lineages recombine with each other, exchanging substantial fraction of their DNA in individual mixed infections [49]. The size of replacement in one event can be as short as 28 bp [50], with the result that linkage is broken down rapidly. This means that individual nucleotides can rise to high frequencies in specific populations, suggesting that local adaptation can potentially occur on a very exquisite scale.

Many of the highly differentiated amino acid changes are close to critical residues of the protein and are plausible candidates to cause important functional changes, based on 3D modelling and previous functional analyses. We have suggested possible functional consequences but validation by targeted experiments and clinical observations is necessary. Although the functional consequences of genomic differentiation of *H. pylori* within different parts of the world remain to be elucidated, the presence of this differentiation already has potential clinical utility. All else being equal, individuals who are infected by *H. pylori* that are characteristically found in high gastric cancer incidence regions are likely to be at higher risk than those associated with lower incidence regions, firstly because the bacteria may be more virulent but secondly because infection with the bacteria might also be a marker for exposure to environmental factors that underlie the high disease risk.

## Supplementary Data

Supplementary material 1

Supplementary material 2

Supplementary material 3

Supplementary material 4

Supplementary material 5
